# Improving treatment for acute ischemic stroke—Clot busting innovation in the pipeline

**DOI:** 10.3389/fmedt.2022.946367

**Published:** 2022-08-01

**Authors:** Joanna Shu Ting Liu, Yiran Ding, Simone Schoenwaelder, Xuyu Liu

**Affiliations:** ^1^Heart Research Institute, The University of Sydney, Sydney, NSW, Australia; ^2^Faculty of Medicine and Health, School of Medical Sciences, The University of Sydney, Sydney, NSW, Australia; ^3^Faculty of Science, School of Chemistry, The University of Sydney, Sydney, NSW, Australia

**Keywords:** thrombolysis (for acute ischaemic stroke), tissue plasminogen activator, anticoagulants, antiplatelets, PROTACs, tick-derived salivary proteins/peptides, targeted degradation, PI3K

## Abstract

Acute ischemic stroke is a consequence of disrupted blood flow to the brain, caused by thrombosis—the pathological formation of occlusive clots within blood vessels, which can embolize distally to downstream tissues and microvasculature. The highest priority of stroke treatment is the rapid removal of occlusive clots and restoration of tissue perfusion. Intravenous thrombolysis is the pharmacological standard-of-care for the dissolution of blood clots, wherein thrombolytic drugs are administered to restore vessel patency. While the introduction of recombinant tissue-plasminogen activator (rtPA) in 1996 demonstrated the benefit of acute thrombolysis for clot removal, this was countered by severe limitations in terms of patient eligibility, lytic efficacy, rethrombosis and safety implications. Development of safer and efficacious treatment strategies to improve clot lysis has not significantly progressed over many decades, due to the challenge of maintaining the necessary efficacy-safety balance for these therapies. As such, rtPA has remained the sole approved acute therapeutic for ischemic stroke for over 25 years. Attempts to improve thrombolysis with coadministration of adjunct antithrombotics has demonstrated benefit in coronary vessels, but remain contraindicated for stroke, given all currently approved antithrombotics adversely impact hemostasis, causing bleeding. This *Perspective* provides a brief history of stroke drug development, as well as an overview of several groups of emerging drugs which have the potential to improve thrombolytic strategies in the future. These include inhibitors of the platelet receptor glycoprotein VI and the signaling enzyme PI3-Kinase, novel anticoagulants derived from hematophagous creatures, and proteolysis-targeting chimeras.

## A brief history of stroke treatment

Stroke is the 2nd leading cause of death and long-term disability worldwide. Acute ischemic stroke (AIS) accounts for 85% of all strokes and is a consequence of pathological blood clotting (thrombosis) occluding vessels supplying the brain. The disruption of blood flow to cerebral tissue rapidly leads to depletion of oxygen and nutrient supply. During stroke, 1.9 million neuronal cells are lost per minute. Moreover, every hour that treatment is delayed, and the occlusive clot persists is estimated to equate to 3.6 years of aging. Annually, ~16 million strokes occur worldwide, causing over 5.5 million deaths. Stroke additionally has a huge socioeconomic and financial impact, causing a loss of ~$100 billion per year in projected healthcare costs and lost productivity.

The development of pharmacotherapies for AIS to rescue blood supply to the brain was primarily inspired by fibrinolysis, which describes an endogenous process of clot removal after clot formation in response to vessel injury, wherein the endogenous serine protease tissue plasminogen activator (tPA) initiates fibrin degradation through catalytic conversion of plasminogen to plasmin. Plasmin mediates the degradation of fibrin polymers that play an important role in stabilizing the platelet thrombus, thereby promoting blood clot dissolution and vascular reperfusion.

Recombinant tPA (rtPA) was first synthesized *via* recombinant DNA technologies by Genentech in 1982, with the aim to amplify the endogenous fibrinolytic pathway. rtPA interacts with surface-bound fibrin on the thrombus to activate plasminogen and amplify fibrin dissolution and clot lysis/removal. rtPA was successfully employed in the treatment of renal vein thrombosis (1) as well as acute myocardial infarction (AMI) (2), these studies demonstrating a restoration of perfusion in humans and dogs respectively. It was not until a landmark study in human patients with AIS (3), when rtPA was approved for clinical use in 1996 by the United States Food and Drug Administration, where it was found to greatly improve functional stroke outcomes based on the National Institutes of Health Stroke Scale (NIHSS) and modified Rankin Scale (mRS) for neurologic disability.

Since 1982, several variations/iterations of rtPA have been generated, differing in activity, half-life, fibrin-specificity, and efficacy- including alteplase, tenecteplase, and reteplase. Alteplase remains the gold standard for clinical thrombolytic therapy and is utilized most readily worldwide (4). On the other hand, the single-chain deletion variant reteplase, has been shown to be better suited toward restoration of coronary patency in AMI (5). Although demonstrating lesser fibrin specificity than alteplase, reteplase has a greater half-life (~18 mins) (6), and can be administered *via* a double bolus (7). This reduced fibrin binding specificity is also suggested to allow reteplase to disengage and infiltrate the clot more freely. Tenecteplase, historically approved for AMI, has enhanced specificity for fibrin, an extended half-life compared to other rtPA alternatives (~22 mins), decreased binding affinity for PAI-1 (with increased resistance), and more practically, can be administered as a single bolus (8). It has been the focus of numerous comparative studies to alteplase (9), as well as recent clinical trials [NCT04797013 {as described in Li et al. (10)}, NCT04071613]. However, it has yet to attain FDA approval, despite its off-label use for intravenous thrombolysis (IVT). Further details can be found here: Chester et al. (4).

## Reperfusion therapy for ischemic stroke

Of highest priority for stroke treatment is removal of the blood clot without causing downstream embolism, leading to reopening (*recanalization*) of occluded vessels, and restoration of blood flow to cerebral tissue (*reperfusion*), the latter strongly predictive of a favorable outcome. Two primary methods are employed to achieve recanalization/reperfusion: IVT and endovascular thrombectomy (EVT) (11). Patient suitability to these procedures is limited by strict assessment criteria including stroke onset, duration, and symptoms, absence/presence of salvageable tissue- verified by imaging (computerized tomographic angiography and magnetic resonance imaging), and the geographical location of patients (regional vs. major city).

IVT refers to the intravenous administration of a thrombolytic drug, given with the aim of clot lysis to recanalize blood vessels and prevent further ischemic damage to tissues and organs. For over 25 years, rtPA has remained the sole approved therapeutic for IVT in AIS. This is despite numerous limitations, namely extensive exclusion criteria (12) and limitations on the administration time window (within 4.5 h stroke onset) (13), along with its potential to increase risk of intracranial hemorrhage (ICH) in 6–7% of patients (14)).

In contrast, EVT is achieved *via* x-ray directed mechanical clot retrieval through insertion of a catheter and stent. It is the more effective therapeutic treatment strategy (15), however is only applied to large-vessel occlusion strokes (LVO-stroke), estimated to make up 24–46% of stroke cases (16). Another drawback for EVT is the requirement of medical infrastructure, equipment, and trained personnel, which is often lacking in non-metropolitan areas.

Despite decades of research, a single clot-busting drug remains available for the treatment of stroke, and its performance for LVO-stroke is poor [15–30% success (17, 18)]. Patients with LVOs who are fortunate enough to receive timely mechanical clot removal are likely to attain better outcomes. However, only an estimated 27% of these patients who attain successful reperfusion after EVT are disability-free at 90 days, with a suspected role for incomplete microcirculatory reperfusion contributing to these suboptimal clinical benefits (19). Although tremendous efforts have been afforded to improve treatment options and overcome barriers to reduce the time for obtaining stroke treatment, ultimately only a small proportion of stroke sufferers receive optimal therapy.

## Improving stroke therapy—Ongoing problems with adjunctive thrombolytics

Current IVT guidelines advise the use of early rtPA administration (up to 4.5 h) based on clinical studies demonstrating improvement in thrombus resolution within this time frame (20). However, under strict eligibility criteria, only about 10% of AIS patients receive rtPA clinically, and of these, recanalization is successful in only 40–50% (21). One major “side-effect” of thrombolysis is the release of clot-bound thrombin upon cleavage of fibrin polymers, which can in turn stimulate further platelet activation and thrombin generation, leading to re-thrombosis in ~34% of cases (22).

In the past two decades, a great deal of effort has been afforded into the development of adjunctive antithrombotics to counter the procoagulant state developed during rtPA administration. Due to the successful use of antithrombotic therapies in the treatment of AMI (for example heparin and aspirin in coronary fibrinolysis) (23), similar approaches have been tested for AIS. Administration of heparin has also been shown to be somewhat beneficial in improving functional outcomes (24, 25). However, more recent studies of heparin therapy in stroke, both in the presence of EVT and/or IVT, show no promising efficacy beyond rtPA alone, with evidence of increased bleeding at moderate heparin doses (MR CLEAN-MED) (26, 27).

The direct thrombin inhibitor (DTI) argatroban has also been shown to enhance the clot-lysis potential of IVT in microcirculation and prevent vascular reocclusion in animal stroke models (28, 29). Its anticoagulation activity has also been extensively studied in patients with heparin-induced thrombocytopenia (HIT) syndrome and demonstrated to be a safe and effective alternative for heparin. A pilot study, ARTSS (Argatroban with tPA for Stroke Study), evaluated the safety of rtPA in combination with argatroban, with significant improvement in complete or partial recanalization reported. However, symptomatic and asymptomatic ICH occurred in 4.6 and 23% of patients respectively, with 10.8% mortality within the first 7 days as a result of extensive hemorrhagic infarction in the brain (30). These studies have highlighted the problems associated with exacerbation of hemorrhagic transformation when employing existing anticoagulation therapy for AIS in combination with rtPA.

In addition to anticoagulants, ample evidence exists to suggest that adjunctive antiplatelet therapy improves recanalization and decreases reocclusion events (31). This is further supported by studies demonstrating that platelet-rich clots are more resistant to tPA-mediated lysis (32). However, like anticoagulants, all currently approved antiplatelets are also unable to preserve the hemostatic balance, and severely increase the risk of ICH when used in the acute setting with IVT. An example of this is tirofiban, a platelet glycoprotein (GP)IIbIIIa (integrin αIIbβ3) receptor antagonist, which demonstrated fatal bleeding prognoses and a death rate of 8% (33, 34). A similar occurrence was observed with the GPIIbIIIa inhibitor abciximab, which reportedly increased ICH risk by 6 times (35).

Additional studies have continued to titrate the doses of these antithrombotics in an attempt to find a safer combination therapy, investigating argatroban and the GPIIb/IIIa-specific antiplatelet eptifibatide (integrilin) (CLEAR-FDR—Study of the Combination Therapy of rtPA and Eptifibatide to Treat Acute Ischemic Stroke, NCT01977456) (36), which was determined safe to proceed to Stage 3 trials. Another notable ongoing trial is MOST [Multi-arm Optimization of Stroke Thrombolysis] (NCT03735979), investigating if argatroban or eptifibatide improve functional stroke outcomes (assessed via modified Rankin scores) compared to gold standards and placebos, with an estimated 2023 completion. A summary of adjunctive alteplase used together with various antithrombotic agents can be viewed in detail in (33).

In summary, current pharmacological reperfusion therapies (IVT) continue to be limited by lack of efficacy, re-thrombosis, and a short therapeutic treatment window. The application of anticoagulant or antiplatelet agents, whilst effective in facilitating thrombolysis and enhancing the rates of successful recanalization in AMI trials, is still confounded by the increased risk of hemorrhagic transformation in the brain. Therefore, a significant push in stroke research is to identify new therapeutic interventions that work synergistically with rtPA to facilitate clot dissolution, whilst maintaining an adequate safety profile- namely preservation of hemostasis. Additional efforts to target inflammation and neuronal toxicity are also ongoing and will not be discussed herein.

Though this *Perspective* focuses on facilitating IVT, it is important to note emerging thrombolytics and treatment strategies can also be applied to EVT, as these techniques are commonly used in tandem clinically. For instance, adjunct therapies also appear promising in the treatment of large vessel occlusions (LVOs). This has been shown in Phase 2 trials of tenecteplase against alteplase, where the former demonstrated improved recanalization and functional grading in LVO patients undergoing EVT (37).

An extended view of promising clinical trials can be found here: (38).

## Promising druggable targets in platelets and clinical trials

Despite advances in antithrombotic therapy, the major issue remains—how can pathological thrombosis be targeted without exacerbating bleeding, particularly in the setting of the brain? This unmet clinical need and commercial gap persists to this day, despite significant research efforts underway to improve adjunct treatments for thrombolysis and stroke treatment.

### GPVI

A promising antiplatelet approach may lie in targeting the GPVI platelet receptor, which is crucially involved in the interaction of platelets with collagen and additionally performs as an important adhesion receptor for fibrin (39). More recently, studies have demonstrated that stroke patients express more GPVI (40), highlighting the potential of targeting platelet buildup *via* this receptor in stroke. In its best recognized role, dimerized GPVI binds collagen and regulates the stabilization of platelet adhesion and eventual thrombus formation. GPVI-deficient individuals have a reduced or limited bleeding propensity (41, 42), though these people are extremely rare. Mice deficient in GPVI have further demonstrated protection against thromboembolism (43), suggesting GPVI to be a promising antiplatelet target. Furthermore, irreversible GPVI abrogation by the monoclonal antibody (mAb) JAQ1 in mice confers protection against thrombosis, however results in receptor shedding and acute thrombocytopenia.

A GPVI fusion protein—Revacept—has recently been investigated in human Phase 2 clinical trials. This protein was shown to indirectly inhibit platelet function through prevention of collagen binding. These studies demonstrated that Revacept reduced arterial thrombosis, embolization, and plaque rupture without impeding hemostasis (44). It was also shown to inhibit platelet interaction with von Willebrand factor through blocking collagen access at the vascular injury site—downregulating platelet adhesion and aggregation (45). However, whilst Revacept reduced thrombosis and conferred less bleeding, whether it has potential to improve rtPA-mediated thrombolysis remains to be investigated. Another GPVI mAb, Glenzocimab (ACT017) (46) has recently completed Phase 1/2a clinical trials (NCT03803007), demonstrating good safety, tolerability and dose escalation data. These studies are now recruiting for Phase 2b/3 trials (NCT05070260), investigating IVT with rtPA within 4.5 h of stroke onset.

### PI3K inhibitors

A successful antithrombotic approach, at least in preclinical settings, has been the targeting of molecules and secondary messengers involved in platelet signaling pathways that activate the major platelet integrin α_IIb_β_3_. An example of this is phosphoinositide (PI) 3-kinase (PI3K), which is downstream of integrin α_IIb_β_3_ (outside-in platelet signaling) and platelet P2Y_12_ receptor-signaling. In this context, PI3K (predominantly *via* p110 beta isoform) (47), plays an important role in sustaining the high affinity state of integrin α_IIb_β_3_ for fibrinogen (48). Inhibition of PI3Kβ reduces integrin α_IIb_β_3_ adhesion and dynamic clot formation, in both *in vitro* and *in vivo* models of thrombosis (49)—however, this is not accompanied by additional hemorrhagic risk, favorably presenting PI3Kβ as a potentially safe therapeutic target for designing anti-platelet drugs.

AZD6482 is a selective and reversible PI3Kβ inhibitor which has undergone several Phase 1 human clinical trials demonstrating antiplatelet efficacy, with no adverse effects on bleeding (50, 51). It has moreover been demonstrated to be safer when combined with other antiplatelets when compared to dual antiplatelet therapy (52). However, the efficacy and safety of AZD6482 as an adjunct to IVT for use in stroke has yet to be determined, with Phase 2a trials scheduled for late 2022 (NCT05363397).

### Drugs derived from hematophagous creatures

Salivary proteins of hematophagous animals are broadly recognized as a rich source of antithrombotic leads that possess low toxicity and immunogenicity and other privileged attributes that are hard to design using small-molecule approaches. It is therefore not surprising to witness the increasing influx of these natural products into clinical trials, with the archetypal anticoagulant hirudin (derived from *Hirudo medicinalis*) and its structural analogs, i.e., bivalirudin and lepirudin, being extensively used clinically for a variety of thromboembolic disorders arising from abnormal thrombin generation.

The activity and function of thrombin are tightly regulated by two positively-charged exosites (I and II) that flank the active site (53). Exosite I acts as the binding site of fibrinogen, positioning it to the active site, which is followed by proteolysis to form fibrin, whilst heparin binds to exosite II and subsequently recruits antithrombin III to irreversibly inhibit the activity of thrombin. Many natural inhibitors of thrombin, i.e., hirudin, hijack both exosite I and the active site to effectively terminate fibrinogen binding and clot formation. A new class of bivalent thrombin inhibitors has been recently developed based on the exosite I and active-site binding scaffolds of variegin derived from the tropical bont tick *Amblyomma variegatum* (54). Variegin has improved affinity (*K*_*i*_ = 277 pM), selectivity, and almost double the *in vivo* half-life compared to that of the standard-of-care bivalirudin–a therapeutic analog of hirudin–(*K*_*i*_ = 1780 pM) (55). Through iterative optimization, ultravariegin was developed as a lead analog possessing 445-fold (*K*_*i*_ = 4 pM) greater inhibitory activity than bivalirudin with 1,000,000-fold selectivity for thrombin over other clotting factors (54). Importantly, variegin/ultravariegin also demonstrated improved ability to preserve the hemostatic capacity, providing three to seven-fold wider therapeutic windows in rodent thrombosis and bleeding models as compared to unfractionated heparin (UFH) and bivalirudin. When used in combination with dual antiplatelet therapy (aspirin and ticagrelor) in a porcine model of stent thrombosis, variegin/ultravariegin significantly reduced the thrombotic potential with a five to seven-fold lower bleeding time than UFH/bivalirudin. Nevertheless, further studies are required to determine if this class of bivalent thrombin inhibitors can be applied adjunctively with rtPA in the treatment of acute coronary syndromes and AIS.

Recently, Payne and co-workers identified another new class of bivalent thrombin inhibitors derived from the bush tick *Haemaphysalis longiconis* (53) that exert their potent inhibitory activity by blocking the active site and exosite II simultaneously, with post-translational sulfation at two conserved tyrosine residues providing significant improvement in the thrombin inhibitory activity. The anticoagulation efficacy of these privileged scaffolds (termed madanin-1 and chimadanin) has been validated in multiple clotting assays, shedding new light on the rational design of peptide substitutes of heparin to prevent the cause of HIT. However, their role in preservation of hemostatic capacity in animal models remains to be determined.

### Proteolysis targeting chimeras (PROTACs)

Studies of knockout animals have substantially enhanced our understanding of the signaling convergence and divergence in hemostasis and thrombosis and provide an important index to predict the safety and efficacy of therapeutic inhibition of target proteins. However, effective techniques for conditional protein removal in adult animals are still urgently required especially for studying embryonic lethal proteins.

**Figure 1:**
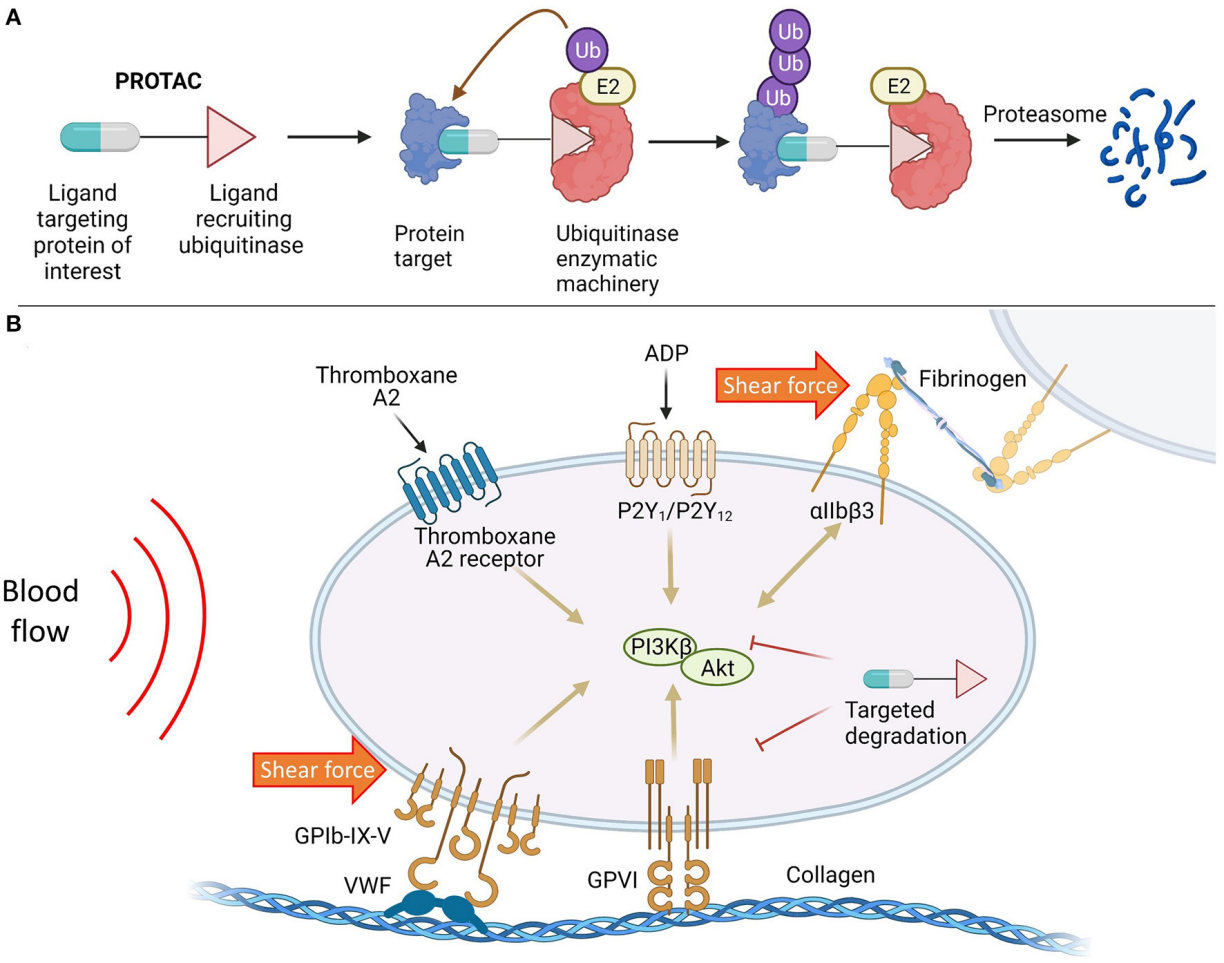
**(A)** Targeted protein degradation mediated by PROTAC molecules; **(B)** Targeted protein degradation as a potential therapeutic strategy to modulate platelet activity in response to biochemical stimulation and shear force.

Recently, a new class of molecules called PROteolysis TArgeting Chimeras (PROTACs) has been described that enable the controlled degradation of specific protein targets for applications in therapeutic discovery (56). PROTACs are bifunctional molecules that simultaneously bind a protein of interest (POI) and a ubiquitinating enzyme (called an E3 ligase) to generate a ternary complex, leading to selective ubiquitination of the POI and degradation through the proteosome (Figure 1A). Unlike gene knockout, this chemical approach is capable of degrading target proteins without the need for genetic manipulation, preserving the integrity of the genome– and is thus especially suitable for studying the pathophysiological functions of embryonic lethal proteins and treatment of acute conditions (i.e., AIS) and short-term illness. Rao and co-workers demonstrated that protein targets can be degraded systematically and quickly (24–72 h) in living animals, including pigs and rhesus monkeys, showcasing the cost-effectiveness and time-efficiency of this methodology for phenotypic characterization in live animals (57). PROTACs have also been utilized to target pro-inflammatory and procoagulant mediators involved in stroke, as demonstrated in (58), where therapeutic degradation of BET proteins enhanced neuroprotection.

At present, PROTAC molecules are mainly deployed to combat cancers and neurodegenerative diseases due to their high efficacy and selectivity over classic inhibitors (59). However, targeted protein degradation has the potential to provide safer and more ethical method of protein depletion in studies of thrombosis/stroke and serves as a novel alternative to CRISPR/Cas9 and Cre/lox systems, enabling target depletion to be accomplished in a time-efficient manner. Furthermore, this platform technology may find broad utility in therapeutic innovation that targets promising antiplatelet approaches, or any potential proteins biasing toward thrombosis with less bleeding risks (Figure 1B), wherein the PROTAC molecule invoking endogenous degradation machineries can be an effective therapeutic in of itself.

## Conclusion

The development of safer and more effective adjunct treatments for stroke is dependent upon the identification of the “holy grail” of antithrombotics, the so-called magic bullet that can provide the balance between efficacy and safety. While emerging technologies will undoubtedly identify increasing targets to pursue in this endeavor, there are several are several promising candidate targets and emerging drugs that have been identified with potential to fulfill this role. Therapies that can dampen the platelet activation, whilst maintaining a level of functionality to preserve haemostasis, including inhibitors of PI3Kb and GPVI, are currently being (or about to be) put through their paces in Phase 2 clinical trials as adjunct therapies for thrombolytic treatment of acute ischaemic stroke. Further to this, novel anticoagulant molecules found in “mother nature” that can neutralize thrombin have also shown potential, and some (i.e., variegin and ultravariegin) have entered comprehensive preclinical assessment. Critically, future clinical evaluation of any emerging antithrombotics must consider their safety when utilized in conjunction with existing therapies, as the majority of stroke patients take antiplatelet and/or “blood-thinning” medications. While we await the outcomes of these and other important clinical trials, it is hoped that one or more of these, alone or combined, may facilitate the safe removal of stroke-causing clots and prevent rethrombosis, leading to improved patient outcomes and reduced mortality in a similar way to that already employed for coronary thrombolysis. Whilst difficult to predict the frontrunner in this pursuit, when identified, this “magic bullet” (or bullets) will ultimately represent a game changer for patients suffering from ischaemic stroke, with potential to improve the survival rates and quality of life for a significant number of stroke sufferers worldwide.

## Data availability statement

The original contributions presented in the study are included in the article/supplementary material, further inquiries can be directed to the corresponding author/s.

## Ethics statement

Ethical review and approval was not required for this study in accordance with the local legislation and institutional requirements.

## Author contributions

JSTL contributed to manuscript writing, editing, and formatting. YD was involved in figure editing and formatting. SS and XL contributed to conceptualization and writing, and actively revised this manuscript. All authors contributed to the article and approved the submitted version.

## Conflict of interest

The authors declare that the research was conducted in the absence of any commercial or financial relationships that could be construed as a potential conflict of interest.

## Publisher's note

All claims expressed in this article are solely those of the authors and do not necessarily represent those of their affiliated organizations, or those of the publisher, the editors and the reviewers. Any product that may be evaluated in this article, or claim that may be made by its manufacturer, is not guaranteed or endorsed by the publisher.
